# A Potential Serum Biomarker for Screening Lung Cancer Risk in High Level Environmental Radon Areas: A Pilot Study

**DOI:** 10.3390/life11111273

**Published:** 2021-11-21

**Authors:** Narongchai Autsavapromporn, Pitchayaponne Klunklin, Imjai Chitapanarux, Churdsak Jaikang, Busyamas Chewaskulyong, Patumrat Sripan, Masahiro Hosoda, Shinji Tokonami

**Affiliations:** 1 Division of Radiation Oncology, Department of Radiology, Faculty of Medicine, Chiang Mai University, Chiang Mai 50200, Thailand; pklunklin@gmail.com (P.K.); imjai.chitapanarux@cmu.ac.th (I.C.); 2 Toxicology Section, Department of Forensic Medicine, Faculty of Medicine, Chiang Mai University, Chiang Mai 50200, Thailand; churdsak.j@cmu.ac.th; 3 Division of Oncology, Department of Internal Medicine, Faculty of Medicine, Chiang Mai University, Chiang Mai 50200, Thailand; busyamas.chewask@cmu.ac.th; 4 Research Institute for Health Science, Chiang Mai University, Chiang Mai 50200, Thailand; patumrat.sripan@cmu.ac.th; 5 Graduate School of Health Science, Hirosaki University, Hirosaki, Aomori 036-8564, Japan; m_hosada@hirosaki-u.ac.jp; 6 Institute of Radiation Emergency Medicine, Hirosaki University, Hirosaki, Aomori 036-8564, Japan; tokonami@hirosaki-u.ac.jp

**Keywords:** radon, serum biomarker, lung cancer, CEA, Cyfra21-1

## Abstract

Radon is a major cause of lung cancer (LC) deaths among non-smokers worldwide. However, no serum biomarker for screening of LC risk in high residential radon (HRR) areas is available. Therefore, the aim of this study was to determine diagnostic values of serum carcinoembryonic antigen (CEA), cytokeratin 19 fragment (Cyfra21-1), human epididymis protein 4 (HE4), interleukin 8 (IL-8), migration inhibitory factor (MIF), tumor nuclear factor-alpha (TNF-α) and vascular endothelial growth factors (VEGF) occurring in high radon areas. Seventy-five LC non-smoker patients and seventy-five healthy controls (HC) were enrolled in this study. Among the HC groups, twenty-five HC were low residential radon (LRR) and fifty HC were HRR. Significantly higher (*p* < 0.0004) serum levels of CEA, Cyfra21-1, IL-8 and VEGF were found in the LC compared with the LRR and HRR groups. More importantly, significantly higher levels (*p* < 0.009) of serum CEA, Cyfra21-1 and IL-8 were observed in HRR compared with the LRR group. Likewise, a ROC curve demonstrated that serum CEA and Cyfra21-1 could better distinguish LC risk from HRR groups than IL-8. These results indicated that serum CEA and Cyfra21-1 were significantly increased in the HRR group and may be considered as potential biomarkers for individuals at high-risk to develop LC.

## 1. Introduction

Lung cancers (LC) are the most aggressive malignant solid tumor causes of cancer-related deaths for both men and women worldwide. Approximately 15–20% of LCs are small cell lung cancers (SCLCs) and other 80–85% of LC are non-small cell lung cancer (NSCLCs). NSCLC can be subdivided into three histological subtypes, namely squamous cell carcinoma, adenocarcinoma and large-cell carcinoma. The treatment of LC includes surgery, chemotherapy and radiation therapy [1,2]. LC progresses quietly and the majority of LC patients are typically diagnosed at an advanced or late stage, with only 15% of LC patients begin diagnosed at an early stage [3]. The median survival of LC patients after treatment is only about 1 year (or less) and the 5-year survival rate is approximately 20% [1]. Over 70% of LC patients are diagnosed in advanced stages because there remains no practical way to identify high-risk individuals. Thus, detection of LC at an early stage could help to improve the survival, prediction of prognosis and treatment outcome of LC patients.

In Chiang Mai province, in upper northern Thailand, LC is the second most common cancer in both men and women, according to the World Health Organization (WHO) Report in 2020 [4]. The main factors identified as responsible for increased LC incidence rate were demographic characteristics, tobacco smoke, secondhand smoke, environmental exposure and indoor radon exposure [5,6,7]. It is considered that 3–20% of all LC deaths worldwide are attributable to indoor radon [8]. Radon (^222^Rn) is a radioactive noble gas from the decay product of uranium-238 (^238^U). It has a half-life of 3.82 days and possesses the capacity of damaging respiratory epithelium cells through the emission of an alpha particle (high linear energy transfer radiation). Radon is present in rock, soil, groundwater, natural gas, and building materials found in dwellings [8,9]. Residential radon exposure depends not only on factors related to housing, but also on the geological structures, the ventilation of radon in air and environmental conditions [10]. According to the WHO, exposure to high levels of radon for a long period of time is the second most common risk factor for LC after tobacco smoke and the major risk factor for LC in non-smokers [8,9]. In addition, the latency of LC is between 5 and 25 years for indoor radon exposure [11]. Thus, long-term exposure to radon and its decay products within dwellings could play an important role in LC risk during a lifetime of exposure in both non-smokers and smokers.

In our previous study, the concentration of indoor radon in Chiang Mai province (57 Bq/m^3^) was considerably higher than the worldwide average value of 39 Bq/m^3^. Within the district of San Pa Tong, the indoor radon activity concentration reached 219 Bq/m^3^, exceeding the WHO reference level of 100 Bq/m^3^ [8]. The annual effective dose was found to be 5.5 mSv, a value higher than the global average of 1 mSv [12]. Therefore, the identification of a useful biomarker for screening the early-stage LC in high residential radon exposure is particularly important for improving LC prognosis and treatment outcomes in Chiang Mai province.

To date, serum biomarkers represent the non-invasive blood test for the screening of LC. Several serum tumor markers for LC have been studied extensively, such as carcinoembryonic antigen (CEA), cytokeratin 19 fragment (Cyfra21-1), human epididymis protein 4 (HE4), interleukin 8 (IL-8), migration inhibitory factor (MIF), tumor nuclear factor-alpha (TNF-α) and vascular endothelial growth factor (VEGF) [2,13,14,15,16,17,18]. However, there is currently no serum biomarker specifically for the detection of LC risk in environmentally high radon areas. Therefore, it is crucial to explore potential serum biomarkers that can detect the diagnosis of LC induced by high radon exposure. In this study, we investigated the serum levels of CEA, Cyfra21-1, HE4, IL-8, MIF, TNF-α and VEGF in LC patients and residential radon exposure, and we evaluated the diagnostic ability of those serum for LC risk in high radon areas.

## 2. Materials and Method

### 2.1. Study Area

**Figure 1:**
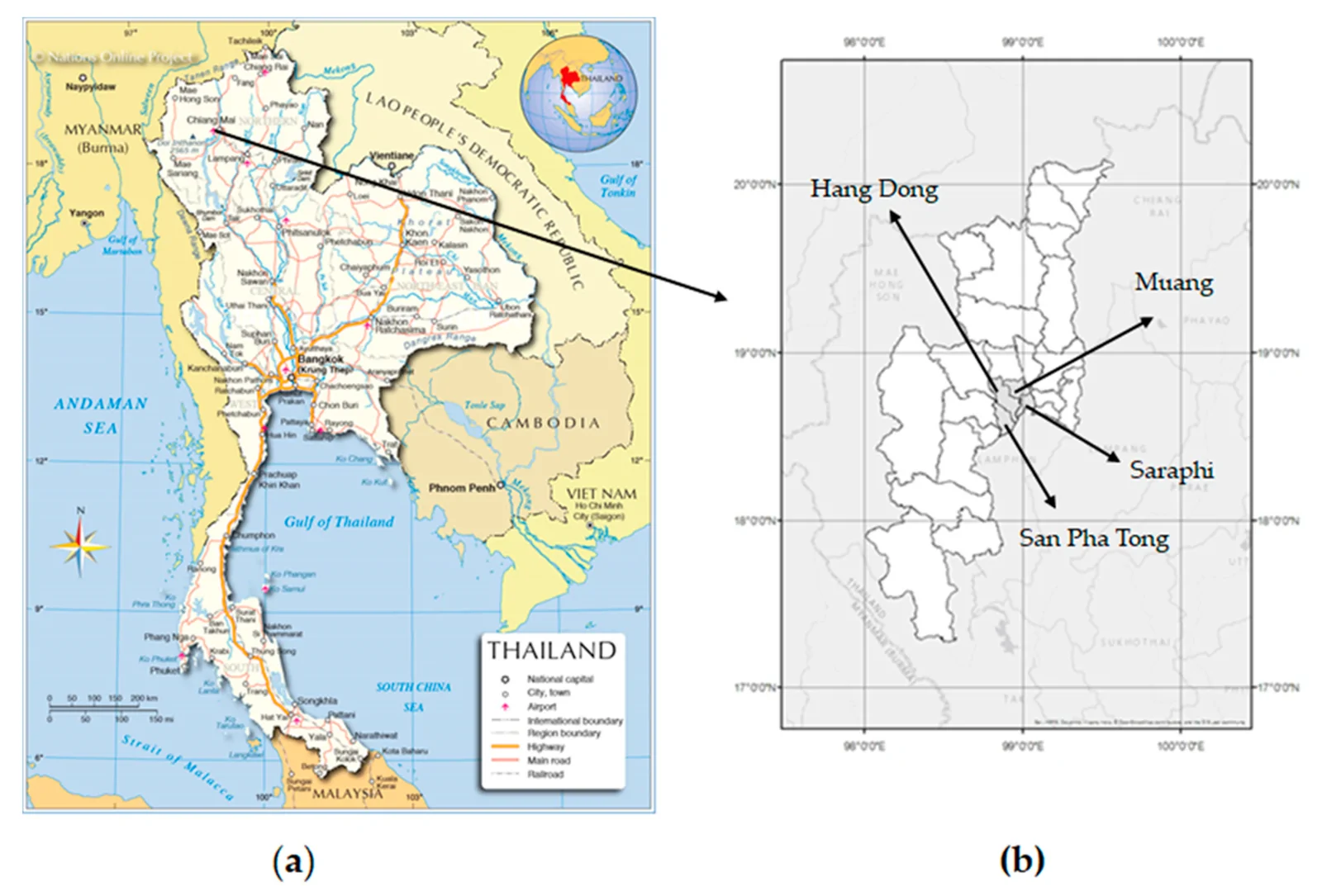
Geological map of Thailand. (**a**) Map of Thailand; (**b**) The study area location in Chiang Mai. Geological map of Thailand obtained from the Nations Online Project (Available online: https://www.nationsonline.org/oneworld/map/thailand_map.htm (accessed on 15 September 2021).

Thailand is a country located in the middle of mainland south-east Asia (Figure 1a). It has a total area of 198,120 square miles with a population of 68 million people [19]. It is bounded to the north by Myanmar and Laos, to the west with the Andaman Sea and Myanmar, to the east by Cambodia and Laos, and to the south by the Gulf of Thailand and Malaysia. Thailand has 77 provinces that are further divided into six geographical regions —Northern, Northeast, Central, Eastern, Western and Southern Thailand—based on natural features: Thailand has a tropical climate, characterized by monsoons [20]. Chiang Mai is the largest city in the upper northern region of Thailand. It is located on the Ping River and surrounded by the mountain ranges of the Thai highlands whose geological and geochemical characteristics increase the levels of natural background radiation from sources such as radon. The city is subdivided into 25 districts. The Hang Dong, Muang, Saraphi and San Pha Tong districts of Chiang Mai were selected as the study area based on the higher mortality rate of lung cancer in upper northern Thailand than in other areas [5,6]. Based on our previous study, the radon levels in the study area are divided into three groups (Figure 1b): “low” (<44 Bq/m^3^), “moderate” (44–70 Bq/m^3^) and “high” (>70 Bq/m^3^) based on indoor radon concentration in the dwellings [12].

### 2.2. Study Design

The transitional study was conducted on selected individuals in the following Chiang Mai districts: Hang Dong, Muang, Saraphi and San Pha Tong (Figure 1b). A total of 150 non-smokers was examined including 75 LC patients (38 males and 37 females), aged from 38 to 87 years, with the median age of 60.3 ± 10.8 years, and 75 healthy controls (HC, 38 males and 37 females), aged from 37 to 86 years, with the median age of 59.6 ± 8.3 years (Table 1). The recruitment period of LC patients took place at Maharaj Nakon Chiang Mai University Hospital and Saraphi Hospital, in Chiang Mai between 2016 and 2020 All LC patients were diagnosed as NSCLC and non-smokers or former smokers (never smoked or stopped smoking for more than 15 years). Then, we randomly selected HC groups that comprised 25 low residential radon or LRR areas (15 males and 10 females) and 50 high residential radon or HRR areas (23 males and 27 females), who had lived in during the past 10 years (or more) in the measured dwellings. All HC groups were individuals without a past history of cancer, minor surgery and non-smokers (never smoked or less than 100 cigarettes smoked in his or her lifetime). Participants were interviewed by trained interviewers using a questionnaire that collected information on possible confounding factors (such as smoking status, lifestyle, environmental tobacco smoke, occupational/environmental/medical exposure to radiation and alcohol consumption).

### 2.3. Sample Collection

A 10 mL of blood samples were collected from both LC and HC groups in a serum- separating sterile tubes. The samples were then centrifuged at 3000× *g* for 10 min at 4 °C and stored at −80 °C for further analysis.

### 2.4. Biochemical Analyses

The serum levels of Cyfra21-1, CEA, HE4, IL-8, MIF, TNF-α and VEGF were performed using a Milliplex map kit assay (Millipore, Billerica, MA, USA) according to the manufacturer’s instructions [21]. All samples were analyzed in duplicate with the xPONENT software (Luminex) and expressed in picograms (pg) per milliliter (mL). The intra-assay and inter-assay variabilities were ≤5%.

### 2.5. Statistical Analysis

The statistical analyses were performed with the software Sigma Plot 10 (Systat Software Inc, San Jose, CA USA). The values of serum (CEA, Cyfra21-1, HE4, IL-8, MIF, TNF-α and VEGF) were summarized as mean ± SD. The significance between the two groups were evaluated by Mann-Whitney U test. To determine the diagnostic value of these analyses, the receiver operating characteristic (ROC) curve was plotted and relevant results including the area under the curve (AUC) combined with sensitivity and specificity were estimated. A *p* values < 0.05 were considered as statistically significant.

## 3. Results

### 3.1. Characteristics of LC and HC Groups

Overall, 75 LC patients and 75 from the HC groups were enrolled in this study. Among the HC groups, 25 individuals (33.3%) were from LRR areas and 50 (66.7%) were from HRR areas. All subjects were non-smokers and all LC patients were diagnosed as NSCLC. There were no statistically significant differences between the two groups in the age (*p* = 0.65) or gender. The median age of LC groups at diagnosis was 60.3 ± 10.8 years. In the HC groups, the median age was 59.6 ± 8.3 years. Thirty-eight (50.7%) were males and thirty-seven (49.3%) were females in both LC and HC groups. The detailed information is shown in Table 1.

### 3.2. Levels of Serum Analytes in LC and HC Groups

**Figure 2:**
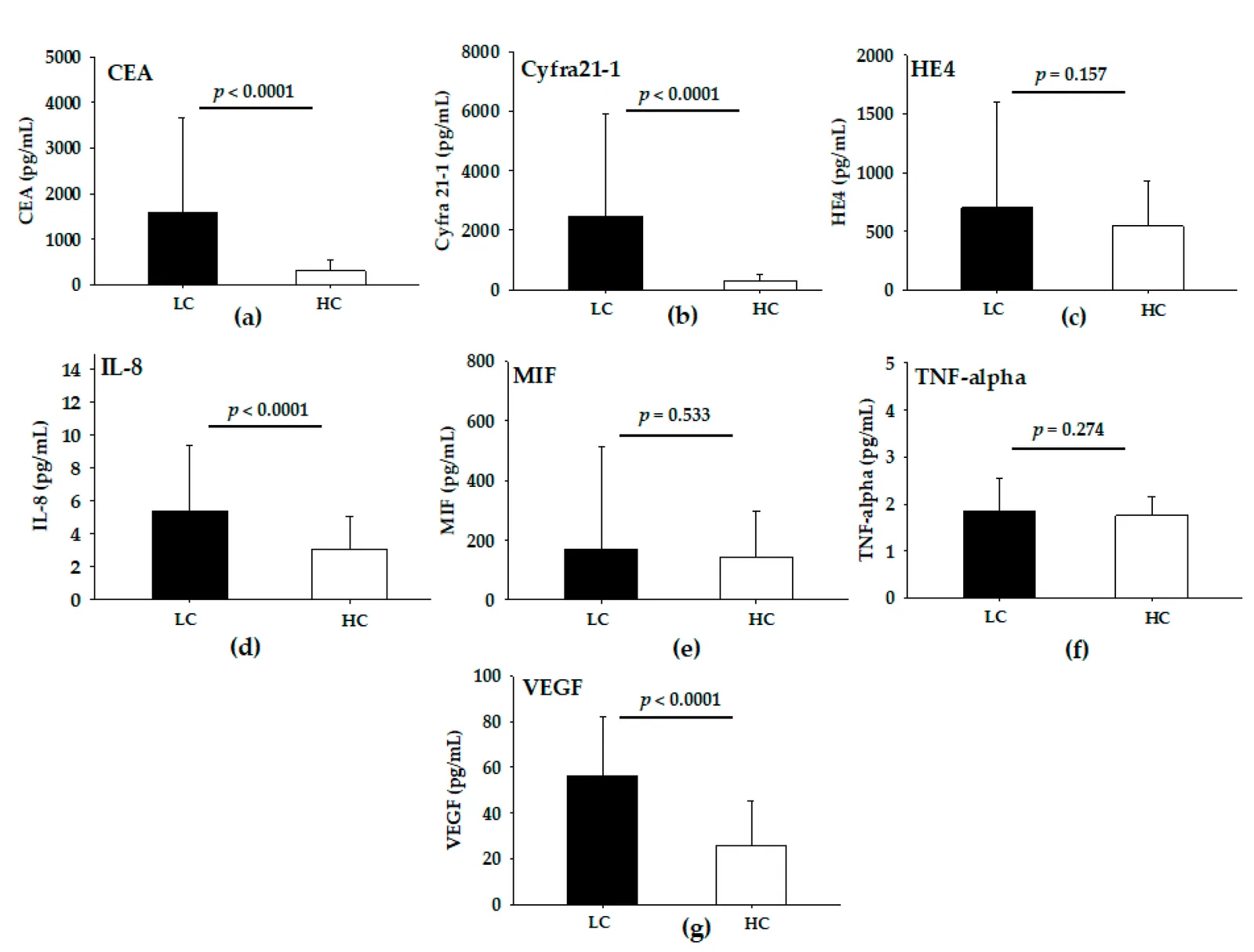
Levels of serum in lung cancer (LC) patients and healthy controls (HC). (**a**) CEA; (**b**) Cyfra21-1; (**c**) HE4; (**d**) IL-8; (**e**) MIF; (**f**) TNF-α; (**g**) VEGF.

The serum levels of CEA, Cyfra21-1, HE4, IL-8, MIF, TNF-α and VEGF in LC and HC groups were presented in Figure 2. The levels of serum CEA, Cyfra21-1, IL-8 and VEGF were significantly significant differences (*p* < 0.0001) between LC and HC groups (Figure 2a,b,d,g). However, no significant differences (*p* > 0.05) were observed in serum HE4, MIF and TNF-α levels between LC and HC groups (Figure 2c,e,f). These results illustrate that serum CEA, Cyfra21-1, IL-8 and VEGF are potential biomarkers for detection of LC risk in HC groups as well as residential radon exposure.

### 3.3. Levels of Serum Analytes in LC, LRR and HRR Groups

**Figure 3:**
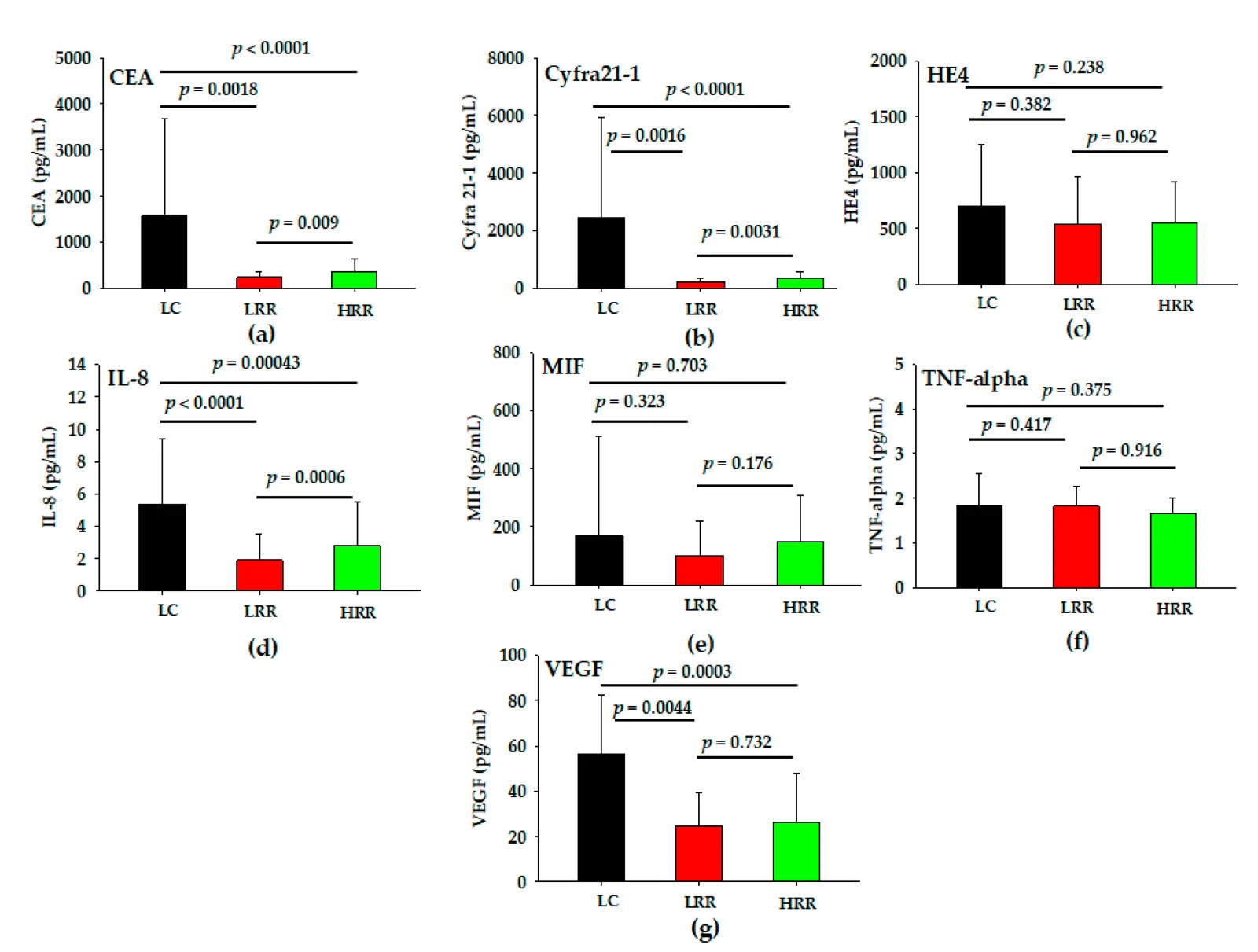
Levels of serum in lung cancer (LC) patients, low residential radon (LRR) and high residential radon (HRR). (**a**) CEA; (**b**) Cyfra21-1; (**c**) HE4; (**d**) IL-8; (**e**) MIF; (**f**) TNF-α; (**g**) VEGF.

To further verify the potential serum biomarker for screening LC risk in high radon areas, the HC groups were divided into LRR and HRR groups according to the radon concentration in their dwellings. As shown in Figure 3, significantly higher (*p* < 0.05) serum levels of CEA, Cyfra21-1, IL-8 and VEGF were observed for the LC group in a comparison between LRR and HRR groups. However, there were no statistically significant differences (*p* > 0.05) in serum HE4, MIF and TNF-α. Furthermore, the levels of serum CEA, Cyfra21-1 and IL-8 were significantly higher (*p* < 0.05) in HRR than LRR groups, but there were no statistically significant differences (*p* > 0.05) between LRR and HRR groups for serum HE4, MIF, TNF-α and VEGF. These results indicated that serum CEA, Cyfra21-1 and IL-8 possess potential ability to distinguish high risk of LC from HC groups.

### 3.4. Diagnostic Ability of Serum Biomarker for LC Risk in High Level Environmental Radon Areas

**Figure 4:**
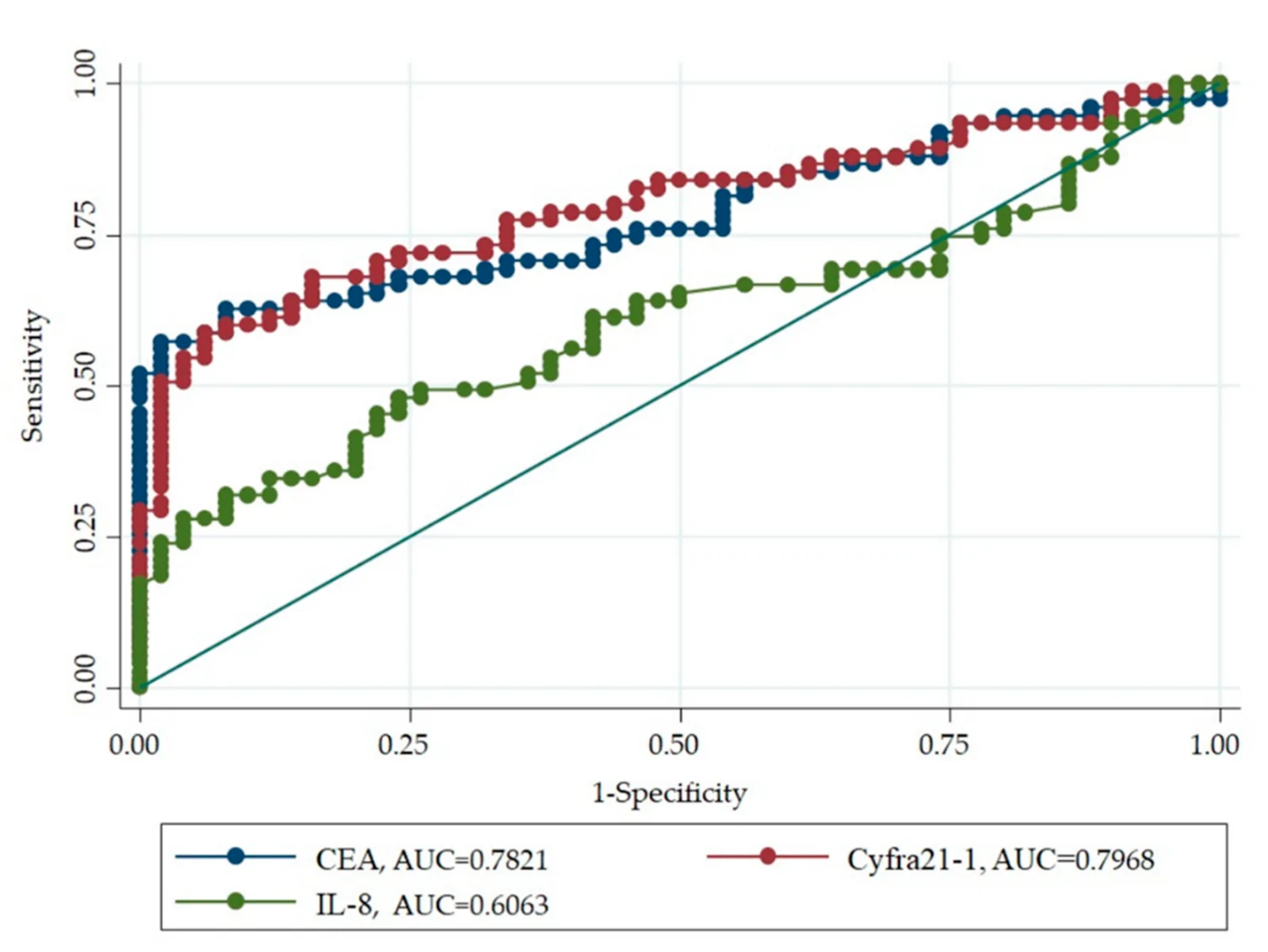
ROC curves for the diagnosis of LC risk in LC patients compared to HRR groups.

After having confirmed that serum CEA, Cyfra21-1 and IL-8 could be better biomarkers to distinguish between LRR and HRR groups, the predictive power as a screening tool to distinguish LC risk from HRR groups was then evaluated. For this purpose, the ROC curves were calculated the diagnostic efficacy of serum CEA, Cyfra21-1 and IL-8 as potential biomarkers of LC risk in high level environmental radon areas. The area under the ROC (AUC-ROC) curve, sensitivity, specificity and all cut-off values of serum were determined using ROC analysis and summarized in Table 2. The AUC-ROC curve for discriminating LC from HRR groups were 0.782, 0.797 and 0.606 for serum CEA, Cyfra21-1 and IL-8, respectively, relative to the HRR groups (Figure 4). The comparison of ROC demonstrated that serum CEA and Cyfra21-1 performed better in identifying LC risk in HRR groups compared with IL-8. Then, we evaluated the sensitivity and specificity of serum CEA, Cyfra21-1 and IL-8 levels in LC patients compared to HRR groups. The sensitivity of serum CEA, Cyfra21-1 and IL-8 were 57.3%, 58.6% and 48% and the specificity were 98%, 94% and 76%. The cut off values of serum CEA Cyfra21-1 and IL-8 were 890.4 pg/mL, 682.5 pg/mL and 5 pg/mL (Table 2). Based on this result, it seems that serum CEA and Cyfra21-1 were better diagnostic markers for early detection of LC risk in high radon areas.

## 4. Discussion

According to the global cancer statistical analysis, LC is one of the main health problems worldwide, showing the highest rates of incidence and death and being the most common cancer among the population in Chiang Mai (Thailand) [1,2,4]. Radon is the seconding leading cause of LC after tobacco smoking and the major risk to non-smokers [5,6,7,8,9,11]. In a previous study we demonstrated that the values of indoor radon concentration in Chiang Mai were considerably higher than the corresponding global average values (39 Bq/m^3^), ranging between 35 to 219 Bq/m^3^, with an average value of 57 Bq/m^3^ [12]. It has been considered that the risk of LC development is increased by 16% per 100 Bq/m^3^ [8,11]. Since the high risk of developing LC is due to long-term toxic effects of radon and its decay products in HRR group, early diagnosis is vital for the prevention, diagnosis and treatment of LC. Our previous study showed that short telomere length and high level of expression of PARP1, WT1, TRERF1 and NOLOC4 serve as biomarkers to screen populations with high risks of LC in high radon exposure areas [12,22]. However, neither method is practical for early screening of LC risk for a large-scale general population. Therefore, finding serum biomarkers as a noninvasive diagnostic method and more rapid technique would improve the diagnosis and treatment of LC for a larger population. There appear to have been no previous studies of serum biomarkers that can be used as LC biomarkers in areas subject to high radon levels.

We evaluated the serum CEA, Cyfra21-1, HE4, IL-8, MIF, TNF-α and VEGF in all non-smoking LC patients with NSCLC and HC groups. In addition, HC groups were divided into LRR and HRR groups according to the radon activity concentration recorded in their dwellings. The results show that serum levels of CEA, Cyfra21-1, IL-8 and VEGF in LC patients were significantly higher (*p* < 0.0001) than in HC groups. This finding is in agreement with previous studies [13,15,16,23,24,25,26]. Further markers such as HE4, MIF and TNF-α are likely not useful as serum biomarkers for detection of LC within this study. The reason for this fact may be due to the different clinical stage, histologic type and smoking status [14,16,17,18,24]. Furthermore, the results showed that serum CEA, Cyfra21-1, IL-8 and VEGF in LC were higher (*p* < 0.05) than in the LRR and HRR groups. Interestingly, significantly higher levels (*p* < 0.05) of serum CEA, Cyfra21-1 and IL-8 were observed in HRR compared with LRR groups. This indicates that a high level of serum CEA, Cyfra21-1 and IL-8 in HRR groups may be a better biomarker of LC risk for differentiating between LRR and HRR groups.

In this study, we also evaluated the diagnostic criteria for predicting LC risk in LC patients compared to HRR groups based on sensitivity, specificity and ROC for serum CEA, Cyfra21-1 and IL-8. We found that the respective sensitivity and specificity were as follows: 57.3% and 98% for CEA; 58.6% and 94% for Cyfra21-1 and 48% and 76% for IL-8. It appears that serum CEA and Cyfra21-1 levels are more accurate, sensitive and specific than that of IL-8. These results further indicated that serum CEA and Cyfra21-1 had a relatively high ability to distinguish LC risk in HRR groups. In addition, the AUC value of serum CEA and Cyfra21-1 were 0.7821 and 0.7968, respectively, and further confirm the ability of these serum to have diagnostic value for LC risk in HRR groups. Based on the findings reported here, this study is the first to establish that serum CEA and Cyfra21-1 were able to select high-risk individuals with LC in high level radon areas, thus having the potential biomarkers to aid in the early screening and diagnosis of those at high-risk of LC. However, these serum markers are relatively limited due to their inadequate sensitivity (~57.3–58.6%). Thus, combined detection of serum CEA, Cyfra21-1 and other markers may improve the early diagnostic sensitivity and decreased specificity, which can lead to faster detection of high-risk groups. These will be the purpose of our future study to provide and improve the evidence for this study.

Nevertheless, a few limitations should be considered when interpreting of research results of this study. Firstly, only gender, age, histologic type and smoking status were included in this study, while other factors such as stage of cancer, alcohol consumption, genetic factors, lung disease, estrogens and occupational/environmental/medical exposure to radiation were not further studied. Secondly, since the sample size was limited, our findings may not be generalizable to other populations. Thirdly, due to the limited number of non-smoking LC patients in the study area, we were not able to divide the group into LC-LRR and LC-HRR groups. However, the results of previous studies have shown that the telomere length, protein expression [12,22] were different in LC patients compared to LRR and HRR groups and similarly our current study also found difference in serum biomarkers among those groups. Finally, this is a preliminary observational study to determine serum CEA and Cyfra21-1 as biomarkers for the diagnosis of LC risk in HRR groups; more longitudinal studies are needed to evaluate and validate the prognostic values in HRR groups with LC and to confirm these findings.

## 5. Conclusions

In summary, the results of the current study show that serum levels of CEA, Cyfra21-1, IL-8 and VEGF were significantly higher in LC patients than residential radon exposure (LRR and HRR groups). Among those biomarkers, serum CEA and Cyfra21-1 performed better in identifying LC risk in HRR groups with satisfactory specificity and sensitivity according to the AUC-ROC. These may be considered as potential serum biomarkers for indicating individuals at high-risk to develop LC. However, further studies in a larger population sample using multiple serum markers are necessary to confirm our current data before serum CEA and Cyfra21-1 can be used clinically as a tumor biomarker for the risk of high radon exposure-induced LC.

## Figures and Tables

**Table 1:** Characteristics of lung cancer (LC) patients and healthy controls (HC).

Characteristics.	LC (*n* = 75)	HC		
		LRR (*n* = 25)	HRR (*n* = 50)	Total (*n* = 75)
Age in years, mean (SD)	60.3 (10.8)	61.2 (7.1)	58.8 (8.8)	59.6.(8.3)
Gender				
Male	38	15	23	38
Female	37	10	27	37

**Table 2:** The diagnostic sensitivity and specificity of serum CEA, Cyrfra21-1 and IL-8 in LC patients compared to HRR groups.

Biomarker	Sensitivity (%)	Specificity (%)	AUC
CEA	57.3	98	0.7821
Cyfra21-1	58.6	94	0.7968
IL-8	48	76	0.6063

## Data Availability

All data are available in this article.

## References

[1] Sung H., Ferlay J., Siegel R.L., Laversanne M., Soerjomataram I., Jemal A., Bray F. (2021). Global cancer statistic 2020: GLOBOCAN estimates of incidence and mortality worldwide for 36 cancers in 185 countries. CA Cancer J. Clin..

[2] Zhao H., Shi X., Liu J., Chen Z., Wang G. (2014). Serum Cyfra21-1 as a biomarker in patients with nonsmall cell lung cancer. J. Can. Res. Ther..

[3] Mao Y., Yang D., He J., Krasna M.J. (2016). Epidemiology of lung cancer. Surg. Oncol. Clin. N. Am..

[4] Thailand-Global Cancer Observatory. https://gco.iarc.fr/today/data/factsheets/populations/764-thailand-fact-sheets.pdf.

[5] Wiwatanadate P. (2011). Lung cancer related to environmental and occupational hazards and epidemiology in Chiang Mai, Thailand. Gene Environ..

[6] Rankantha A., Chitapanarux I., Pongnikorn D., Prasitwattanaseree S., Bunyatisai W., Sripan P., Traisathit P. (2018). Risk patterns of lung cancer mortality in northern Thailand. BMC Public Health..

[7] Reungwetwattana T., Oranratnachai S., Puataweepong P., Tangsujarivijit V., Cherntanomwong P. (2020). Lung cancer in Thailand. J. Thorac. Oncol..

[8] World Health Organization (2009). Handbook on Indoor Radon.

[9] United Nations (2010). Scientific Committee on the Effect of Atomic Radiation. Source and Effects of Ionizing Radiation: United Nations Scientific Committee on the Effect of Atomic Radiation: UNSCEAR 2008 Report to the Genera; Assembly with Scientific Annexes.

[10] Sabbarese C., Ambrosino F., D’Onofrio A. (2021). Development of radon transport model in different types of dwellings to assess indoor activity concentration. J. Environ. Radioact..

[11] Al-Zoughool M., Krewski D. (2009). Health effects of radon: A review of the literature. Int. J. Radiat. Biol..

[12] Autsavapromporn N., Klunklin P., Threeatana C., Tuntiwechapikul W., Hosada M., Tokonami S. (2018). Short telomere length as a biomarker risk of lung cancer development induced by high radon levels: A pilot study. Int. J. Environ. Res. Public Health.

[13] Dong Y., Zheng X., Yang Z., Sun M., Zhang G., An X., Pan L., Zhang S. (2016). Serum carcinoembryonic antigen, neuron-specific enolase as biomarkers for diagnosis of nonsmall cell lung cancer. J. Can. Res. Ther..

[14] Zeng Q., Liu M., Zhou N., Liu L., Song X. (2016). Serum human epididymis protein4 (HE4) may be a better tumor marker in early lung cancer. Clin. Chim. Acta.

[15] Tas F., Duranyildiz D., Oguz H., Camlica H., Yasasever V., Topuz E. (2006). Serum vascular endothelial growth factor (VEGF) and interleukin-8 (IL-8) levels is small cell lung cancer. Cancer Investig..

[16] Balla M.M., Desai S., Purwar P., Kumar A., Bhandarkar P., Shejul Y.K., Pramesh C.S., Laskar S., Pandey B.N. (2016). Differential diagnosis of lung cancer, its metastasis and chronic obstructive pulmonary disease based on serum VEGF, Il-8 and MMP-9. Sci. Rep..

[17] Gámez-Pozo A., Sánchez-Navarro I., Calvo E., Teresa Agulló-Ortuño M.T., López-Vacas R., Díaz E., Camafeita E., Nistal M., Madero R., Espinosa E. (2012). PTRF/Cavin-1 and MIF proteins are identified as non-small cell lung cancer biomarkers by label-free proteomics. PLoS ONE.

[18] Chen Z., Xu Z., Sun S., Yu Y., Lv D., Cao C., Deng Z. (2014). TGF-β1, IL-6 and TNF-α in bronchoalveolar lavage fluid: Useful markers for lung cancer?. Sci. Rep..

[19] National Statistical Office Thailand Population and Housing Census 2019. http://web.nso.go.th/index.htm.

[20] Department of Mineral Resources Geology of Thailand. http://www.dmr.go.th/main.php?filename=Mineral_re2015_EN.

[21] Oyanagi J., Koh Y., Sato K., Mori K., Teraoka S., Akamatsu H., Kanai K., Hayata A., Tokudome N., Akamatsu K. (2019). Predictive value of serum protein levels in patients with advanced non-small cell lung cancer treated with nivolumab. Lung Cancer.

[22] Autsavapromporn N., Dukaew N., Wongnoppavicvich A., Chewaskulyong B., Roytrakul S., Klunklin P., Phantawong K., Chitapanarux I., Sripan P., Kritsananuwat R. (2019). Identification of novel biomarkers for lung cancer risk in high levels of radon by proteomics: A pilot study. Radiat. Prot. Dosimetry.

[23] Hanagiri T., Sugaya M., Takenaka M., Oka S., Baba T., Shigematsu Y., Nagata Y., Shimokawa H., Uramoto H., Takenoyama M. (2011). Preoperative Cyfra21-1 and CEA as prognostic factors in patients with srage I non-small cell lung cancer. Lung Cancer.

[24] Dalaveris E., Kerenidi T., Katsabeki-katsafli A., Kiropoulos T., Tanou K., Gourgoulianis K.I., Kostikas K. (2009). VEGF, TNF-α and 8-isoprostane levels in exhaled breath condensate and serum of patients with lung cancer. Lung Cancer.

[25] Cai D., Xu Y., Ding R., Qiu K., Zhang R., Wang H., Huang L., Xie X., Yan H., Deng Y. (2020). Extensive serum biomarker analysis in patients with non-small-cell lung carcinoma. Lung Cancer.

[26] Balla M.M.S., Patwardhan S., Melwani P.K., Purwar P., Kumar A., Pramesh C.S., Laskar S., Pandey B.N. (2021). Prognosis of metastasis based on age and serum analytes after follow-up of non-metastatic lung cancer patients. Transl. Oncol..

